# Angiotensin-converting enzyme gene insertion/deletion polymorphism in migraine patients

**DOI:** 10.1186/1471-2377-8-4

**Published:** 2008-03-26

**Authors:** Erling Tronvik, Lars J Stovner, Gunnar Bovim, Linda R White, Amanda J Gladwin, Kathryn Owen, Harald Schrader

**Affiliations:** 1 Department of Neurosciences, Norwegian University of Science and Technology, Trondheim, Norway; 2 Department of Neurology and Clinical Neurophysiology, University Hospital of Trondheim, Trondheim, Norway; 3 AstraZeneca, R&D Genetics, Mereside, Alderley Park, Macclesfield, Cheshire, SK10 4TG, UK

## Abstract

**Background:**

The main objective of this study was to investigate the angiotensin converting enzyme (*ACE*) genotype as a possible risk factor for migraine (both with and without aura) compared to controls. We also wanted to examine whether a clinical response to an ACE inhibitor, lisinopril, or an angiotensin II receptor blocker, candesartan, in migraine prophylaxis was related to *ACE *genotype.

**Methods:**

347 migraine patients aged 18–68 (155 migraine without aura (MoA), 187 migraine with aura (MwA) and 5 missing aura subgroup data) and 403 healthy non-migrainous controls > 40 years of age were included in the study. A polymerase chain reaction (PCR) was performed on the genomic DNA samples to obtain the *ACE *insertion (I)/deletion(D) polymorphisms.

**Results:**

No significant differences between migraine patients and controls were found with regard to *ACE *genotype and allele distributions. Furthermore, there was no significant difference between the controls and the MwA or MoA subgroups.

**Conclusion:**

In our sample there is no association between *ACE *genotype or allele frequency and migraine. In addition, *ACE *genotype in our experience did not predict the clinical response to lisinopril or candesartan used as migraine prophylactics.

## Background

Two small open studies reported an improvement of the headache in migraine patients using an angiotensin-converting enzyme (ACE) inhibitor [1,2]. Indirectly, a beneficial effect of angiotensin II receptor blockers (ARB's) on headache is shown in a meta-analysis on side effects reported in placebo controlled trials including over 12 000 patients [3]. Two randomized, placebo controlled studies conducted by our research group have evidence for efficacy of an ACE inhibitor (lisinopril) and an ARB (candesartan) in migraine prophylaxis [4,5]. This and other evidence points in the direction of involvement of the renin-angiotensin system (RAS) in migraine pathophysiology. (For further discussion on possible mechanisms see reference [6]).

The human angiotensin converting enzyme (*ACE*) gene consists of either an insertion (I) allele or a deletion (D) allele forming three possible genotypes: II, ID or DD. Many studies have suggested an association between the *ACE-D *allele and cardiovascular diseases [7]. For migraine an Italian (Paterna) [8], an Australian (Lea) [9], and a Japanese (Kowa) [10] study has demonstrated different results regarding whether an association between the *ACE *polymorphisms and this condition exists (Table 1).

**Table 1:** *ACE *genotype and allele distributions among controls and migraine patients in different studies

		**Genotypes**			**Alleles**	
	**N**	**DD(%)**	**ID(%)**	**II(%)**	**D(%)**	**I(%)**

**Controls**						
Tronvik	403	92 (26.6)	204 (50.6)	107 (22.8)	388 (48.1)	418 (51.9)
Paterna (ref 8)	201	75 (37.3)	101 (50.3)	25 (12.4)	251 (62.4)	151 (37.6)
Lea (ref 9)	244	76 (31.1)	122 (50.0)	46 (18.9)	274 (56.1)	214 (43.9)
Kowa (ref 10)	248	31 (12.5)	114 (46.0)	103 (41.5)	176 (35.5)	320 (64.5)
**Migraine**						
Tronvik	347	78 (22.5)	186 (53.6)	83 (23.9)	342 (49.3)	352 (50.7)
Paterna	302	146 (48.3)	129 (42.7)	27 (9.0)	421 (69.7)	183 (30.3)
Lea	250	77 (30.8)	142 (56.8)	31 (12.4)	296 (59.2)	204 (40.8)
Kowa	176	33 (18.7)	86 (48.9)	57 (32.4)	152 (43.2)	200 (56.8)
**MwA subgroup**						
Tronvik	155	34 (21.9)	87 (56.1)	34 (21.9)	155 (50.0)	155 (50.0)
Paterna	NA	NA	NA	NA	NA	NA
Lea	151	48 (31.8)	85 (56.3)	18 (11.9)	181 (59.9)	121 (40.1)
Kowa	54	14 (25.9)*	26 (48.2)	14 (25.9)	54 (50.0)*	54 (50.0)
**MoA subgroup**						
Tronvik	187	43 (23.0)	96 (51.3)	48 (25.7)	182 (48.7)	192 (51.3)
Paterna	302	146 (48.3)*	129 (42.7)	27 (9.0)	421 (69.7)	183 (30.3)
Lea	99	29 (29.3)	57 (57.6)	13 (13.1)	115 (58.1)	83 (41.9)
Kowa	122	19 (15.6)	60 (49.2)	43 (35.2)	98 (35.2)	146 (59.8)

* Reported significant finding for genotype or allele frequencies

The objectives of the present study were two-fold. Firstly we wanted to examine whether a beneficial effect in the above mentioned migraine prophylactic studies [4,5] could be predicted by *ACE *genotype, a question that has also been raised in a recent publication [11]. Secondly we wanted to investigate the *ACE *genotype as a possible risk factor for migraine with (MwA) and without (MoA) aura in a Norwegian population.

## Methods

Included in the study were 347 migraine patients aged 18–68 (155 MwA, 187 MoA and 5 missing aura subgroup data, based on ICHD-2 criteria [12]) and 403 healthy non-migrainous controls > 40 years of age. The migraineurs were recruited partly from the lisinopril [4] (n = 49) and candesartan [5] (n = 59) studies, and the remaining group (n = 239) from the outpatient clinic of the Department of Neurology, Trondheim University Hospital. The patients and the controls were recruited from the same area and only subjects with Nordic ethnic background were included. The diagnosis was confirmed by an experienced clinical neurologist. Responder status in the candesartan and lisinopril studies was defined as a reduction in days with headache of at least 50% in the treatment period compared to the placebo period. Non-responders were the subjects not defined as responders and with both genotype and response data available. No patients were included in both the lisinopril and candesartan studies. The control group was recruited in collaboration with the Department of Immunology and Transfusion Medicine and criteria for inclusion were no present or former history of migraine or other types of chronic headaches, no history of epilepsy or of hypertension in need of medical treatment, and age > 40 years (since status as "non-migraineur" cannot be determined with relative certainty before this age). No direct interview was made in the control group, but the participants filled out a questionnaire to determine eligibility for participation. In addition to not having migraine the control group was required to have no other headache condition and less than one headache day per month.

The migraine group had a mean age of 41 years (standard deviation (SD): ± 12 years) and consisted of 268 women and 79 men. Median age of migraine onset was 16 years and median attack frequency was 4.0 attacks per month. In the control group with 233 women and 170 men, mean age was 50 years (SD: ± 7 years).

311 of the samples were genotyped by AstraZeneca, R&D Genetics, UK, and 439 samples were genotyped at the Department of Neurology, Trondheim University Hospital, Norway.

### Genomic DNA preparation and polymerase chain reaction (PCR) analysis

DNA was extracted from peripheral EDTA-blood stored at -80°C. The D and I alleles were identified on the basis of PCR amplification of the respective fragments from intron 16 of the *ACE *gene. The oligonucleotide primers [13,14] used (MedProbe) were sense (forward): 5' CTGGAGACCACTCCCATCCTTTCT 3' and antisense (reverse): 5' GATGTGGCCATCACATTCGTCAGAT 3'. Amplification was performed with 0.5 μmol of each primer. The PCR product was a 191 bp fragment in the absence, and a 479 bp fragment in the presence of the insertion. Homozygous D alleles were confirmed using the insertion-specific primer 5' TTTGAGACGGAGTCTCGCTC 3'.

Part of the samples (n = 311) were amplified using a thermal cycler and the products separated on 2% agarose gel. The remaining samples (n = 439) were analyzed using a LightCycler instrument (Roche). Amplification conditions for the first method were 1.2 mM MgCl_2_, 1 U AmpliTaq Gold, 200 μM dNTPs and 5 μL DNA template in a total reaction volume of 25 μL, enzyme activation at 94°C for 20 min, denaturation at 94°C for 1 min, annealing at 58°C for 1 min and extension at 72°C for 2 min for a total of 32 cycles. Samples analyzed by LightCycler used the FastStart DNA Master SYBR Green 1 mix, which includes Taq DNA polymerase (Roche Diagnostics), plus 3 mM MgCl_2_, and 2 μL DNA template, in a total reaction volume of 20 μL with enzyme activation at 95°C for 10 min, denaturation at 95°C for 10 s, annealing at 50°C for 5 s, and elongation at 72°C for 15 s, for a total of 35 cycles. The fluorescence intensity of the double-strand specific SYBR Green I is directly proportional to the amount of PCR product formed. Melting curves indicated the respective melting temperatures of the 191 bp and 479 bp fragments to be 84.5°C and 91.8°C respectively, with samples from heterozygotes displaying a peak at both temperatures. Reaction products were confirmed on 2% agarose gel. The ratio between cases and controls was the same for both methods of analysis and blinded control experiments in 10 random patients analysed by the first method were confirmed by the second method.

### Statistical analysis

Observed genotype count was used to calculate genotype and allele frequencies for the *ACE I/D *polymorphism. The expected genotype proportions were calculated and compared to the observed proportions according to the Hardy-Weinberg law. The significance level was set at p < 0.05. For comparison between groups we used the χ^2 ^test with one or two degrees of freedom. To compare means (age of debut, frequency of migraine/headache) we used one-way ANOVA. Statistical analysis were performed using SPSS version 13.0 for Windows (SPSS Inc., Chicago, IL, USA). Power calculation for the association between *ACE *polymorphisms and migraine was performed with the method described by Altman with correction for unequal sample sizes [15]. For the association between *ACE *polymorphisms and drug response, we performed a one sample two tailed test with alpha = 0.05.

### Ethics

The study was approved by the regional committee for ethics in medical research, and by the Norwegian data inspectorate. All subjects included gave a written informed consent.

## Results

The observed genotypes in the control population did not deviate significantly from the Hardy-Weinberg equilibrium (p = 0.98). With regard to the genotype and allele distributions, no significant differences between migraine patients and controls were detected, even though the *ACE-D *allele tended to be more frequent (p = 0.058) among responders than non-responders in the candesartan group (Table 2). Furthermore, there was no significant difference between the controls and the MwA or MoA subgroups, nor between responders and non-responders to lisinopril and candesartan, and no difference was detected when stratifying by sex. Within the migraine group differences in genotype could not explain the presence of aura (n = 342, missing data = 5, p = 0.64), of coexisting tension-type headache among migraineurs (n = 343, missing data = 4, p = 1.0), differences in age of debut (n = 342, missing = 5, p = 0.69) or frequency of migraine (n = 342, missing = 5, p = 0.52) or in headache frequency as recorded in the placebo period in the candesartan study (n = 56, missing = 3, p = 0.77).

**Table 2:** *ACE *genotype and allele distributions among controls and migraine patients in a Norwegian population

		**Genotypes**			**Alleles**	
	**N**	**DD(%)**	**ID(%)**	**II(%)**	**D(%)**	**I(%)**

**Controls**	403	92 (26.6)	204 (50.6)	107 (22.8)	388 (48.1)	418 (51.9)
**Migraine**	347	78 (22.5)	186 (53.6)	83 (23.9)	342 (49.3)	352 (50.7)
**MwA subgroup**	155	34 (21.9)	87 (56.1)	34 (21.9)	155 (50.0)	155 (50.0)
**MoA subgroup**	187	43 (23.0)	96 (51.3)	48 (25.7)	182 (48.7)	192 (51.3)
**Lisinopril responders**	12	2 (16.7)	6 (50.0)	4 (33.3)	10 (41.7)	14 (58.3)
**Lisinopril non-responders**	37	10 (27.0)	16 (43.2)	11 (29.7)	36 (48.6)	38 (51.4)
Candesartan responders*	18	7 (38.9)	9 (50.0)	2 (11.1)	23 (63.9)	13 (36.1)
Candesartan non-responders*	38	8 (21.1)	18 (47.4)	12 (31.6)	34 (44.7)	42 (55.3)
**Responders combined**	30	9 (30.0)	15 (50.0)	6 (20.0)	33 (55.0)	27 (45.0)
**Non-responders combined**	75	18 (24.0)	34 (45.3)	23 (30.7)	70 (46.7)	80 (53.3)

* Response data available in 56 of 59 genotyped

Allele and genotype frequency distributions are not significantly different for any diagnostic groups (migraine, MwA, MoA) vs controls, or for responders vs non-responders (p > 0.05).

Frequencies of the genotypes and alleles for the different studies are presented in Table 1. There are large differences in genotypes and alleles among the controls. E.g. the II genotype varies between 12.4 and 41.5 and the D-allele between 35.5 and 62.4%.

## Discussion

In the present Norwegian sample, there is no difference in *ACE *genotype or allele frequency in a migraine group compared to a control group. Associations between *ACE *polymorphism and migraine reported in other studies are not consistent and have been detected in different diagnostic or sex categories. The results of these studies are shown in Table 1. In addition a recently published study from Taiwan found no differences in *ACE *allelic frequencies between migraine patients and controls, but stratified by gender the DD frequency was significantly lower in male migraineurs than controls (not included in Table 1 because only the abstract was published in the English language) [16]. Findings that the DD genotype is more frequent in MoA [8] and MwA [10] or less frequent in male migraineurs [16] are not supported by our data. Our population which is the largest to date used to study the relationship between *ACE *polymorphism and migraine (MoA and MwA) should have >80% power to detect an association of the same magnitude as in the study by Paterna et al [8]. Our study also did not find a relationship between *ACE *genotype and response to prophylactic drugs influencing the RAS. The allele frequency in the responders versus non-responders in the candesartan group had a p-value of 0.058 and with low numbered groups the risk of a false negative result is present.

The purpose of looking at the association between responders in the two clinical trials and *ACE *genotype was not to detect a small theoretical association, in which case this subgroup analyses would be underpowered, but to see whether there was an association so strong that it would be valuable in clinical use predicting response in migraine – prophylactic treatment. That is whether it would be clinically beneficial to use *ACE *genotype to predict whether the patient would respond to the drug or not. Our opinion is that in order for an association to be clinical valuable at least 75–100% of responders to a migraine-prophylactic drug should have a specific *ACE*-genotype. The power of our study to measure a percentage of 75% DD among the candesartan responders with the control population as reference is > 80%.

A limitation of the study is that the control group was not directly interviewed increasing the risk of migraineurs self-reporting themselves as non-migraineurs and thereby increasing the risk of type II errors. In order to minimize this problem participants in the control group were required to have no other headache condition and less than one headache day per month.

Population stratification refers to differences in allele frequencies between cases and controls due to systematical differences in ancestry rather than in the association of genes with disease [17,18]. There are large differences in the frequencies of the *ACE*-alleles in different populations (Table 1). Hence, due to the problem with population stratification we did not find it meaningful to perform a statistical analysis of the merged data of all these studies. This might have been misleading also because the way diagnosis were made, both of migraineurs and controls, may differ somewhat between the studies.

## Conclusion

There was no difference in *ACE *genotype distribution between a migraine and a control population in our material. Our study also indicates that *ACE *genotyping will not be a valuable tool for predicting clinical response of drugs influencing the angiotensin system in headache treatment. It is, however, important that these findings should be confirmed in other studies with more patients and among different ethnic groups.

## Abbreviations

RAS, renin-angiotensin system; ACE, angiotensin converting enzyme; MoA, migraine without aura; MwA, migraine with aura; ARB's, angiotensin II receptor blockers.

## Competing interests

Co-authors Amanda Gladwin and Katryn Owen are AstraZeneca staff.

No disclosures from the rest of the authors.

## Authors' contributions

ET, LJS, GB, LRW and HS were involved in designing the study. AG, KO and LRW were responsible for the genotyping. ET and LJS conducted the statistical analyses. All authors were involved in either drafting the manuscript or revising it.

## Pre-publication history

The pre-publication history for this paper can be accessed here:


